# Tissue Culture of Oil Palm: Finding the Balance Between Mass Propagation and Somaclonal Variation

**DOI:** 10.3389/fpls.2019.00722

**Published:** 2019-06-04

**Authors:** Sylvie Weckx, Dirk Inzé, Ludo Maene

**Affiliations:** ^1^Deroose Plants NV, Evergem, Belgium; ^2^Center for Plant Systems Biology, VIB, Ghent, Belgium; ^3^Department of Plant Biotechnology and Bioinformatics, Ghent University, Ghent, Belgium

**Keywords:** oil palm (*Elaeis guineensis* Jacq.), tissue culture, somatic embryogenesis, micropropagation, somaclonal variation, mantled abnormality

## Abstract

The oil palm (*Elaeis guineensis* Jacq.) is typically propagated *in vitro* by indirect somatic embryogenesis, a process in which somatic cells of an explant of choice are, via an intermediate phase of callus growth, induced to differentiate into somatic embryos. The architecture of the oil palm, lacking axillary shoots, does not allow for vegetative propagation. Therefore, somatic embryogenesis is the only alternative to seed propagation, which is hampered by long germination times and low germination rates, for the production of planting material. The current oil palm somatic embryogenesis procedure is associated with several difficulties, which are described in this review. The limited availability of explants, combined with low somatic embryo initiation and regeneration rates, necessitate the proliferation of embryogenic structures, increasing the risk for somaclonal variants such as the mantled phenotype. Several ways to improve the efficiency of the tissue culture method and to reduce the risk of somaclonal variation are described. These include the use of alternative explants and propagation techniques, the introduction of specific embryo maturation treatments and the detection of the mantled abnormality in an early stage. These methods have not yet been fully explored and provide interesting research field for the future. The development of an efficient oil palm micropropagation protocol is needed to keep up with the increasing demand for palm oil in a sustainable way. Mass production of selected, high-yielding palms by tissue culture could raise yields on existing plantations, reducing the need for further expansion of the cultivated area, which is often associated with negative environmental impacts.

## Introduction

The African oil palm (*Elaeis guineensis* Jacq.) is a tropical plantation crop cultivated for its production of two types of vegetable oil: palm oil and palm kernel oil. It is a monocotyledonous species belonging to the family of Arecaceae, which includes more than 2,000 palm species. The genus *Elaeis* contains two species: *E. guineensis* or African oil palm and *E. oleifera* or American oil palm. The African oil palm is native to West Africa, but nowadays 84% of palm oil production takes place in Indonesia and Malaysia^1^. Because of its low oil yield, the American oil palm is not cultivated on commercial plantations. However, it has some interesting characteristics, including a slow height increment, resistance to certain diseases and oil enriched in unsaturated fatty acids, making it an interesting candidate for hybridization with the African oil palm. The resulting interspecific hybrid *E. oleifera* × *E. guineensis* is mainly cultivated in South America (Hormaza et al., 2012;Sumaryono et al., 2018).

**Figure 1:**
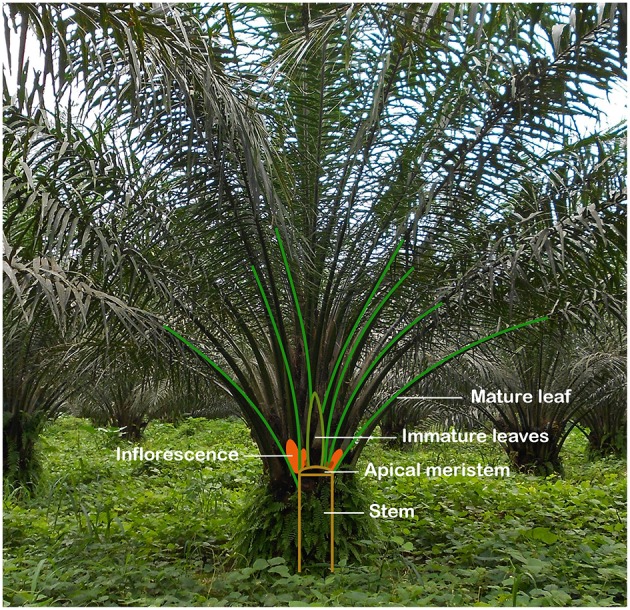
Oil palm tree architecture. The oil palm is built up of a single stem without branches, topped by 40–50 mature leaves. It only has one apical meristem, located below a cylinder of immature leaves, which are used as explants for somatic embryogenesis. Each leaf has an inflorescence meristem in its axil.

**Figure 2:**
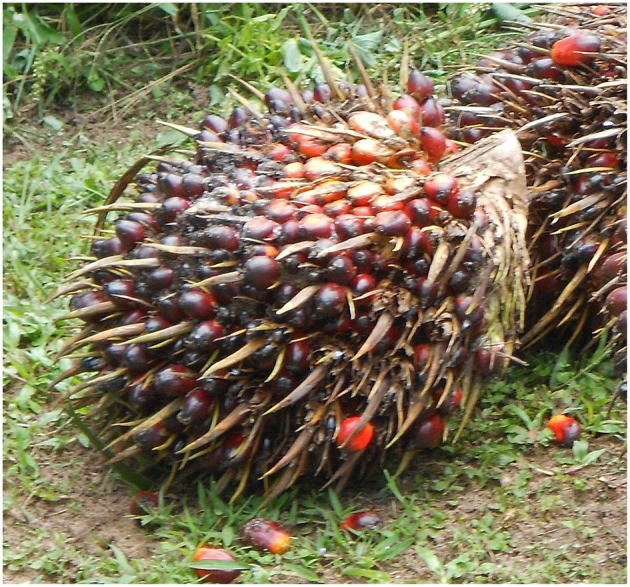
Oil palm fruit bunch. After pollination, female inflorescences develop into fruit bunches consisting of up to 2,000 fruits, which are rich in palm oil and palm kernel oil.

The oil palm has a single vegetative shoot meristem, giving rise to an unbranched stem topped by 40–50 leaves (Figure 1). Each new leaf is accompanied by an inflorescence meristem in its axil, that under appropriate conditions develops into a fruit bunch, containing up to 2,000 fruits (Figure 2). Palm oil is extracted from the outer mesocarp of these fruits, while palm kernel oil is extracted from the inner kernel. Palm oil has a large range of applications. About 90% is used in food industry, e.g., to produce margarine, shortenings, biscuits, ice-cream, salad dressings, mayonnaise etc. The remaining 10% is used for soap and oleochemical manufacturing (Mba et al., 2015). Palm oil currently is the most important vegetable oil worldwide, accounting for 36% of the total vegetable oil production, while palm kernel oil accounts for 4%^1^.

The increased demand for vegetable oils in the last two decades has encouraged governments of developing countries to promote oil palm cultivation. Oil palm cultivation was seen as a way to alleviate poverty, often at the expense of the tropical rainforest (Pirker et al., 2016). Over the last 20 years, the production of palm oil has increased almost fourfold, from 19 million tons in 1998 to 73 million tons in 2018^1^, accompanied with an increase of area from 7 million of hectares in 1998 to 23 million of hectares in 2018^1^. Considering a continuously growing world population and a rise of standard of living in the developing world, palm oil production is expected to increase further to 150 million tons per year in 2050 (Corley, 2009), asking for a sustainable solution. One way to meet the increased demands in a sustainable way, is to improve yields on the current cultivated area, as to avoid the clearance of extra land with high environmental value. Despite oil palm already having a much higher oil productivity than other oil crops (Rival and Levang, 2014), there is plenty of room for yield improvement. Currently, an average yield of 3.13 tons of palm oil ha^−1^ yr^−1^ is achieved^1^, which is far below the potential yield of 10–11 tons ha^−1^ yr^−1^ estimated by Breure (2003).

Oil palm plantations mainly consist of hybrid *tenera* trees, which are produced by crossing a *dura* mother plant and a *pisifera* father plant. The *dura, pisifera*, and *tenera* varieties differ in shell thickness and oil yield. While fruits of the *dura* variety have a relatively thick shell and thin mesocarp, *pisifera* fruits are thin-shelled and have a thick mesocarp, rich in palm oil. However, *pisifera* palms are usually female-sterile, hampering oil production. *Tenera* fruits have an intermediate shell and mesocarp thickness, yielding 30% more than *dura* fruits (Singh et al., 2013a) and are thus the preferred variety for oil production.

As shown on Figure 1, the oil palm does not produce axillary shoots, making vegetative propagation impossible. Around 98% of oil palm planting material therefore consists of hybrid seeds (Kushairi et al., 2010). Tissue culture derived plantlets make up the remaining 2% of planting material. Although its limited implementation, tissue culture provides some benefits over traditional seed propagation. In natural conditions, oil palm seed germination is a very slow process, requiring several years, and germination rates are very low. Procedures, mainly consisting of heat pre-treatments, have been developed to speed up the germination process, but several months are still required to obtain germination and lots of non-germinated seeds are lost (Green et al., 2013). Moreover, genetic improvement by seed propagation is cumbersome. Selection cycles last for around 10 years and high heterogeneity is observed among hybrids (Rival, 2000). Some palms of a certain cross yield 60% more than the average progeny of that cross. Yearly yield increases through breeding of selected oil palms amount on average 1.5% (Woittiez et al., 2017). In contrast, a yield increase of at least 20% could be obtained by cloning high-yielding trees by tissue culture (Kushairi et al., 2010). Moreover, tissue culture can accelerate the multiplication of trees with interesting characteristics related to disease resistance, growth pattern or oil composition. Also, tissue culture is an important tool allowing the regeneration of plantlets modified by modern genetic techniques such as the CRISPR/Cas 9 system (Bortesi and Fischer, 2015), which will become very important for the creation of novel genetic variants in the future.

Traditional tissue culture methods, such as node or meristem cultures, cannot be used for oil palm micropropagation. In these methods a piece of plant tissue containing a pre-existing meristem, namely an axillary bud or shoot apical meristem, is isolated and grown on a culture medium to provide a new shoot. Because the oil palm only has one meristem, it must be propagated *in vitro* by indirect somatic embryogenesis (SE). In this process somatic cells of a plant tissue of choice differentiate, via an intermediate callus phase, into bipolar structures resembling zygotic embryos (von Arnold et al., 2002). The resulting somatic embryos can then germinate into plantlets.

Oil palm tissue culture is not widely implemented because of several difficulties associated with it. These include a low overall efficiency, caused by low somatic embryo initiation and regeneration rates, and a high risk for somaclonal variation. Embryogenic structures are extensively propagated to increase the SE efficiency, thereby increasing the frequency of somaclonal variation (Rival et al., 2013). In this review, we discuss the main difficulties associated with oil palm tissue culture and propose several research strategies to optimize oil palm tissue culture.

## Oil Palm Somatic Embryogenesis

### General Principles of Somatic Embryogenesis

**Figure 3:**
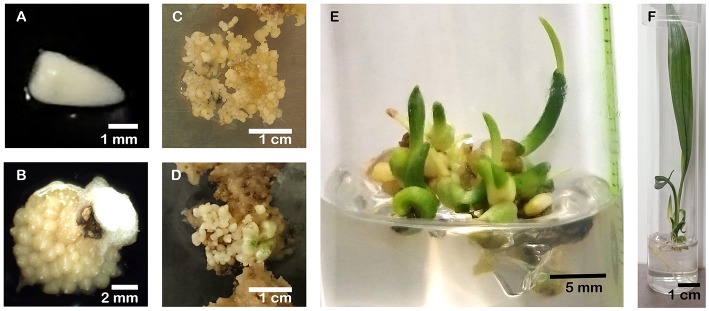
Different steps of indirect somatic embryogenesis from oil palm zygotic embryos. **(A)** Immature zygotic embryos are cultivated on a callus induction medium containing high auxin concentrations to induce cell dedifferentiation. **(B)** After 8 weeks, the formation of primary, nodular callus can be observed. **(C)** After 16 weeks, nodular calli have developed into embryogenic callus, that can be proliferated on semi-solid or liquid proliferation media. **(D)** Embryogenic cells are transferred to a culture medium with reduced auxin concentrations to initiate the formation of somatic embryos. **(E)** After a subsequent maturation phase in which the embryos accumulate storage material and acquire desiccation tolerance, germination starts. **(F)** Plantlets having a well-developed shoot and root system are being formed.

Somatic embryogenesis is defined as a process in which somatic cells dedifferentiate into totipotent embryonic cells, that can further develop into somatic embryos (SEs) (Guan et al., 2016). Like zygotic embryos (ZEs), SEs are characterized by a bipolar structure with typical embryonic organs such as a radicle, hypocotyl, and cotyledons. However, SEs and ZEs follow different developmental pathways (von Arnold et al., 2002; von Arnold, 2008). SE seldomly occurs under natural conditions (Garcês et al., 2014), but it can be induced *in vitro* as a method for multiplication of selected plants by application of appropriate plant growth regulators (PGRs) and growth conditions. Although the potential of SE has been shown for many different plant species, only a few species are propagated at large-scale by SE (von Arnold, 2008). Two types of SE have been reported: direct and indirect SE. In direct SE, SEs are induced directly from the explant, without an intervening callus phase (Guan et al., 2016). It is suggested that direct SE proceeds from embryogenically predetermined cells, such as the hypocotyl epidermis of young plantlets, the nucellus, synergids, or embryonic cells. On the other hand, indirect embryogenesis proceeds from differentiated cells, which are induced to dedifferentiate into callus cells, followed by the acquirement of an embryogenically determined state (Williams and Maheswaran, 1986). Both pathways show many similarities and it is often difficult to make a strict distinction between them (von Arnold et al., 2002). As for many other species, mainly the indirect pathway is followed during oil palm SE. Direct oil palm SE has only been reported once (Jayanthi et al., 2011). The focus in this review will therefore be on indirect SE, which typically includes the following five steps (Figure 3): (1) initiation of embryogenic callus (EC) cultures; (2) proliferation and maintenance of EC; (3) initiation of SEs (also referred to as expression of SE); (4) maturation of SEs; and (5) plantlet regeneration (von Arnold et al., 2002; von Arnold, 2008; Corredoira et al., 2019).

### Oil Palm Somatic Embryogenesis Protocols

Despite that worldwide many researchers are working on the establishment and optimization of protocols for commercial micropropagation of oil palm, no general protocol is available. A large amount of papers focusses on the use of mature and immature zygotic embryos as explants, which are not suitable for commercial purposes. Moreover, much of the research has been carried out by companies with commercial interests, often reporting insufficient details for replication of the protocol (Scherwinski-Pereira et al., 2010). Also, responses depend on the genotype, the age of the parent tree and the type of explant used, requiring adaptation of the protocol to the actual conditions. A representative overview of some oil palm SE protocols available in literature, is shown in Table 1.

**Table 1:** Oil palm somatic embryogenesis protocols.

**Explant type**	**References**	**Phase**	**Growth regulators (μM)**	**Activated charcoal (g/L)**	**Carbohydrate source (g/L)**	**Gelling agent (g/L)**	**Basal medium**	**Environmental conditions**	**Duration and subculture interval**	**Success rate**
Immature zygotic embryo	Wan Nur Syuhada et al., 2016	CI	2,4-D 9.95	0	Sucrose 30	Phytagel 2.5	MS macronutrients + Y3 micronutrients	28°C 50–60 μmol m^−2^s^−1^ 16 hd^−1^ photoperiod	SI: 1 m	Callus: 41.25%
		PC	2,4-D 25			No, suspension culture	MS	28°C, dark		
		SE/M/R	NAA 0.1			Phytagel 2.5		n.i.		
	Thuzar et al., 2011	CI	2,4-D 9.05	0	Sucrose 30	Phytagel 3	N6	27°C 50–60 μmol m^−2^s^−1^ 16 h d^−1^ photoperiod	SI: 3 m	Callus: 81% Embryogenic callus: 32% Regeneration: 56%
		SE/M	2,4-D 0.45	2					3–5m, SI: 1 m	
		R	–	0.5					3–6 m	
Mature zygotic embryo	Balzon et al., 2013; de Carvalho Silva et al., 2014; Gomes et al., 2015, Gomes et al., 2016	CI	Picloram 450	2.5	Sucrose 20–30	Phytagel 2.5	MS	25°C, dark	150 d, SI: 1 m	Embryogenic callus: 97.5% Regeneration: 70% Regeneration efficiency in TIS: 293.7% biomass gain after 90 d Rooting: 92.9%
		PC^(1−3)^	Picloram 40 2-ip 10	0	Sucrose 20-30^(1−2)^				Up to 12 w, no subculture	
		SE/M	2-ip 12.3 NAA 0.54	0–0.3	Sucrose 20–30				90 d, SI: 1 m	
		R	–	0–2.5	Sucrose 20–30	Phytagel 2.5 or no (TIS)	1/2 MS or MS	25°C 38–52 μmol m^−2^s^−1^ 16 h d^−1^ photoperiod	3 subcultures of 30 d	
		RT^(2−3)^	IBA 53.7	0	Sucrose 30	Double-phase: phytagel 2.5 + liquid layer	MS	25°C 50–52 μmol m^−2^s^−1^ 16 h d^−1^ photoperiod	Up to 150 d	
	Monteiro et al., 2017	CI	Picloram 450	2.5	Sucrose 30	Phytagel 2.5	MS	25°C, dark	5 m, SI: 1 m	Callus: 69.3–81.1% Embryogenic callus: 13.1–29.5% Embryogenesis: 32.1–77.2% Regeneration: 8.9–50%
		PC	2,4-D 5	0		No, liquid culture			210 d, SI: 2 w	
		SE/M	–	2.5		Phytagel 2.5	1/2 MS		Up to 12 m	
		R					MS	n.i.	n.i.	
	Kanchanapoom and Domyoas, 1999	CI	2,4-D 9.05	0	Sucrose 30	Gelrite 1.5	Y3	27°C20 μmol m^−2^s^−1^15 h d^−1^ photoperiod	8 w	Embryogenesis: 8.33%
		PC/SE	2,4-D 2.26				1/2 MS		16 w, SI: 8 w	
		M	2,4-D 0.45	0.5					5 w	
		R	BA 11.10						SI: 8 w	
	Teixeira et al., 1995	PC	2,4-D 10	0	Sucrose 15 + glucose 5	No, suspension culture	Y3	26-27°C, dark	SI: bi-weekly	
Immature male inflorescence	Jayanthi et al., 2015	CI	2,4-D 150 Picloram 150	3	Sucrose 30	Agar 8	Y3	27°C, dark	4–6 subcultures of 4 m, with gradual auxin reduction of 50%	Callus: 42–72% Embryogenesis: 6.8–9.35%
		SE/M/R	BA 18 ABA 3.78 GA 5.78					27°C 4,000 lux 16 h d^−1^ photoperiod	n.i.	
		RT	IAA 23 IBA 19.6	0.5	Sucrose 60				n.i.	
Immature female inflorescence	Guedes et al., 2011	CI	2,4-D 225-450	3	Sucrose 30	Phytagel 2.5	MS	25°C, dark	Up to 42 w	Embryogenic callus: 54.8%
	Teixeira et al., 1994	CI	2,4-D 475	3	Sucrose 30	Gelrite 2.2	1/2 MS macronutrients + 1/1 MS micronutrients	27–29°C, dark	81 w, SI: 3–4 w	Callus: 30–50%
		SE/M	NAA 15 ABA 2	0			Y3	n.i.	n.i.	
		R	–	3	Sucrose 20		1/2 MS	n.i.	8 w	
Immature leaf	Hashim et al., 2018	CI	NAA 27–54 2,4-D 0–4.5	0	Sucrose 30	Agar 7	MS	Dark	Up to 1 year, SI: 3 m	40–29,115 shoots produced from a single ortet isolation
		SE/PE/M	Half of CI concentration, followed by a gradual reduction					28°C 12 h d^−1^ photoperiod	Up to 30 m, SI: 2 m	
		R	NAA 0.11			Gelrite 2.5			2–3 m	
		RT	NAA 1.1			No, liquid of double-phase			2–3 m	
	Gomes et al., 2017	CI	Picloram 450	2.5	Sucrose 30	Phytagel 2.5	MS	25°C, dark	Up to 8 subcultures of 30 d	Callus: 22.3% Embryogenesis: 5%
		SE	2-ip 12.3 NAA 0.54	0				25°C 52 μmol m^−2^s^−1^ 16 h d^−1^ photoperiod	6 subcultures of 30 d	
		M	–	2.5		No, TIS			2 subcultures of 30 d	
	Corrêa et al., 2016	CI	2,4-D 800	3	Sucrose 30	Phytagel 2.5	Y3	27°C, dark	90 d	Callus: 0.28–52.05%
		PC	2,4-D 9 Putrescine 1000	0					60 d	
		SE/M	2,4-D 0.1 Putrescine 1000	n.i.					60 d	
		R	NAA 0.54 Putrescine 1,000	0				27°C 40 μmol m^−2^s^−1^ 16h d^−1^ photoperiod	60 d	
	Constantin et al., 2015	CI	NAA 107.41	0	Commercial sugar 50	Agar 9	MS	28°C, dark	SI: 12 w for explant and 8 w once callus is formed	Callus: 1.39–30.56%
		PC/SE/M	Gradual auxin reduction of 25% during each subculture					29°C 1,000 lux	SI: 6 w	
	de Touchet et al., 1991	PC	2,4-D 362-452	1	Glucose 20	No, suspension culture	MS	27°C 50 μmol m^−2^s^−1^ 12 h d^−1^ photoperiod	SI: 4–6 w	4-fold weight increase after 1 month
Young plantlet	Jayanthi et al., 2011	SE	2,4-D 40 2,4,5-T 10 NAA 40 TDZ 10 BA 10	3	Sucrose 30	Agar 8	3	27°C, dark	8 w, SI: 1 m	Direct embryogenesis with a rate of 80–100%
		R	BA 2 ABA 1	n.i.	n.i.	n.i.		27°C 40 μmol m^−2^s^−1^ 16 h d^−1^ photoperiod	n.i.	
	Scherwinski-Pereira et al., 2010	CI	Picloram 450	0.3	Sucrose 30	Phytagel 2.5	MS	25°C, dark	12 w, no subculture	Embryogenic callus: 41.5%
		SE/M	NAA 0.6 2-ip 12.3	0.3					SI: 4 w	
		R	–	1	Sucrose 20		1/2 MS	25°C 35 μmol m^−2^s^−1^ 16 h d^−1^ photoperiod	n.i.	

*CI, callus induction; PC, proliferation of callus; PE, proliferation of embryoids; SE, somatic embryo initiation; M, somatic embryo maturation; R, plantlet regeneration; RT, rooting; n.i, no information provided; SI, subculture interval; d, days; w, weeks; m, months*.

As can be seen in Table 1, mainly two types of auxins are used for the induction of EC, namely 2,4-dichlorophenoxyacetic acid (2,4-D) and picloram. The former is a very popular PGR for induction of SE in many species, used in 49% of the protocols, while the latter is only used in 5% of the protocols (Jiménez, 2005). Picloram is however frequently employed for SE in different palm species, including areca palm (Karun et al., 2004), peach palm (Steinmacher et al., 2007), and açaí palm (de Olivera Freitas et al., 2016). Independent of the type of explant used, high concentrations of auxins are required for EC induction in oil palm. Generally, concentrations fluctuate around 9 to 10 μM in the absence of activated charcoal (AC) and around 450 μM in the presence of AC. Exceptionally, concentrations up to 800 μM have been reported (Corrêa et al., 2016). AC is a very porous compound with a unique absorption capacity, absorbing part of the PGRs, making it difficult to know what concentration is available to the plant. It is frequently added to the culture medium, because it limits the accumulation of phenolics and other toxic compounds by absorbing them (Thomas, 2008). Since oil palm tissue is very susceptible to phenolic oxidation, often 2.5–3 g L^−1^ AC is added to the EC induction medium, but several reports of media without AC have been made as well. The effect of AC on EC induction has been examined by Balzon et al. (2013). Both the percentage of explants with EC as the percentage of coverage of the explants by EC were significantly increased by the addition of 2.5 g L^−1^ AC to the medium. The effect of different auxin treatments on oil palm SE has also been examined, but with contradictory results coming out. Both Scherwinski-Pereira et al. (2010) and Balzon et al. (2013) observed a higher percentage of explants forming EC when using picloram than when using 2,4-D, but the opposite was observed by Thuzar et al. (2011). Jayanthi et al. (2015) did not obtain significant differences in callus percentages between both auxins, but embryogenesis was strongly improved when using a combination of both 2,4-D and picloram.

During the EC proliferation and maintenance phase, auxin concentrations similar to the induction phase are being used to inhibit early development in SEs. While AC is often added to induction media, it is mostly removed from the culture medium when proceeding to the proliferation phase. EC cultures can be maintained for a long time, but the callus may lose its embryogenic capacity after a prolonged culture period and the risk for somaclonal variation increases (von Arnold et al., 2002; Rival et al., 2013), which must be taken in mind when establishing proliferation cultures. Three different set-ups for EC proliferation have been developed: (1) semi-solid cultures (Balzon et al., 2013); (2) suspension cultures (de Touchet et al., 1991; Teixeira et al., 1995; Wan Nur Syuhada et al., 2016; Monteiro et al., 2017); and (3) temporary immersion systems (Sumaryono et al., 2008; Marbun et al., 2015; TIS). All three systems have their pros and cons. About 79% of the oil palm genotypes can be propagated in semi-solid media, compared to only 20% in liquid cultures (Alwee et al., 2010). However, commercial micropropagation based on semi-solid media solely is technically unfeasible: it is very labor-intensive and requires a lot of space, leading to high production costs. Liquid media are easier to handle, bigger vessels are used and cultures develop in a more synchronized way (Gomes et al., 2016). Moreover, the use of liquid media has been shown to stimulate the reactivity of many species in tissue culture (Etienne and Berthouly, 2002), which might be explained by the greater contact between the explant and the medium, facilitating the uptake of water and nutrients, as well as by the dilution of toxic exudates (Monteiro et al., 2017). The difficulties associated with the use of liquid media, namely the lack of oxygen and the risk for hyperhydricity, can be overcome by using TIS. In TIS the plant material is only periodically immersed in culture medium, allowing adequate oxygen provision (Etienne and Berthouly, 2002). The stimulatory effect of suspension cultures on oil palm embryogenesis was shown by Monteiro et al. (2017). Around 52.4% of the calli proliferated in a liquid medium containing 2,4-D formed SEs when plated on a semi-solid medium, compared to only 14% of the calli proliferated on semi-solid media.

For induction and maturation of SEs, EC are transferred to a medium with reduced auxin concentration. Sometimes, the concentration of auxins is gradually reduced with each subculture step (Constantin et al., 2015; Jayanthi et al., 2015; Hashim et al., 2018). The auxins picloram and 2,4-D that are commonly used during the EC initiation phase, are often replaced by the weaker auxin 1-naphthaleneacetic acid (NAA). A small amount of cytokinins, mainly 2-isopentenyladenine (2-ip), is added in a few protocols as well. Since the use of cytokinins is often associated with the occurrence of somaclonal variations (Eeuwens et al., 2002), some efforts have been made in the replacement of cytokinins with polyamines, mainly putrescine, which promote cell growth and DNA stability (Rajesh et al., 2003; Corrêa et al., 2016). A few protocols describe the use of abscisic acid (ABA) to improve embryo maturation (Teixeira et al., 1994; Jayanthi et al., 2015).

In most of the protocols published, SEs are transferred to a medium without any PGRs to obtain plantlet regeneration. Also, the concentration of AC is often increased again in this phase, in order to absorb remaining PGRs that might inhibit germination (Corredoira et al., 2019).

The basic salt composition of the cultivation media generally consists of a full strength Murashige and Skoog (MS) medium (Murashige and Skoog, 1962). The strength of the medium is sometimes reduced to half in the regeneration phase. Some other medium compositions that have been shown to be suitable for oil palm cultivation are the Y3 medium (Eeuwens, 1978), the N6 medium (Chu et al., 1975), or even combinations of these media (Wan Nur Syuhada et al., 2016).

A sucrose concentration of 20–30 g L^−1^ is generally used in all phases. No reports describing the effect of different sucrose concentrations, neither of other carbohydrate sources, on oil palm SE are available. For solidification of the medium, most of the time gellan gum (Gelrite™ or Phytagel™) is added at a concentration around 2.5 g L^−1^. In a few cases agar is used. On top of AC and putrescine, some other additives are frequently added to the culture medium. These include hydrolyzed casein (Kanchanapoom and Domyoas, 1999; Jayanthi et al., 2011, 2015; Thuzar et al., 2011; Corrêa et al., 2016; Gomes et al., 2016, 2017), the amino acids glutamine (Scherwinski-Pereira et al., 2010; Guedes et al., 2011; de Carvalho Silva et al., 2014; Gomes et al., 2016, 2017; Wan Nur Syuhada et al., 2016; Monteiro et al., 2017), asparagine and arginine (Jayanthi et al., 2015; Corrêa et al., 2016), ascorbic acid (Teixeira et al., 1995; Monteiro et al., 2017), and polyvinylpyrrolidone (Teixeira et al., 1994, 1995), added at variable concentrations.

While, with the exception of a few protocols (Kanchanapoom and Domyoas, 1999; Thuzar et al., 2011; Wan Nur Syuhada et al., 2016), cultures are kept in dark conditions during the EC induction phase, in around 50% of the protocols cultures are transferred to light conditions for induction and maturation of SEs. Regeneration is always performed in light conditions, with a photoperiod of 16 h d^−1^ and a photosynthetic photon flux varying between 20 and 60 μmol m^−2^s^−1^. During all phases, temperatures are kept constant, around 25–29°C.

In many cases, explants are transferred to fresh medium with the same composition on a monthly base until EC are observed. Longer subculture intervals have been reported by Kanchanapoom and Domyoas (1999) and Jayanthi et al. (2015), transferring their explants after 8 and 16 weeks, respectively. EC formation without subculture has been reported by Scherwinski-Pereira et al. (2010). The effect of monthly subcultures was examined by Monteiro et al. (2017). He discovered that primary callus formation is not influenced by the subculture treatment, but embryogenic callus formation is. Without subculture only 0.8% of the explants formed EC, compared to 20.8% when subcultures were performed on a monthly base. Regularly transferring explants to fresh medium can thus boost embryogenesis. Also in the next phases of the SE protocol, explants are generally transferred at monthly intervals to fresh medium.

## Problems Associated With Oil Palm Somatic Embryogenesis

### Limited Availability of Explants

Because of the sturdy architecture of the oil palm, there is very limited choice of explants that can be used for initiation of embryogenic cultures. Typically, immature leaf explants are being used for commercial production of tissue culture plantlets (Schwendiman et al., 1988; Constantin et al., 2015; Corrêa et al., 2016), but not without difficulties.

Immature leaves are leaves that have not emerged yet. They are grouped in a cylinder only 6–7 cm above the single apical meristem of the palm, at the center of the canopy (Figure 1) (Hashim et al., 2018). Because the ortet, the parent plant from which the clone descends, is injured during isolation of the leaf cylinder, it can only be sampled once every 3–5 years (Alwee et al., 2010), allowing the tree to recover. In worst cases, the apex is damaged during the isolation, leading to the loss of a valuable ortet. Not only can each ortet be sampled very seldomly, also the number of plantlets that can be regenerated from one isolation is low. When applying only limited EC proliferation in order to reduce the risk for somaclonal variation, 100–10,000 plantlets can be regenerated from a single leaf cylinder isolation. This means that many ortets are necessary to provide a continuous supply of tissue culture plant material (Alwee et al., 2010). Moreover, it is important to mention that all ortets, except in the case of re-cloning, are genetically different, meaning that only a limited amount of plants of a certain genotype can be regenerated.

### Low Efficiency of Somatic Embryogenesis

Somatic embryogenesis generally is known as a process with low efficiencies, yielding only a small number of plantlets. The two main factors limiting oil palm SE are: (1) the initiation of SEs out of EC cultures, and (2) the conversion of SEs in fully developed plantlets.

Depending on the genotype used, between 1 and 52% of immature leaf explants form callus (Corrêa et al., 2016). For other explant types, reported callus formation rates are higher, with up to 97.5% of zygotic embryos (Balzon et al., 2013) and up to 72% of immature inflorescence explants (Jayanthi et al., 2015) reacting. Although many explants are thus capable of regaining totipotency, on average, depending on the genotype, only around 5% of the explants regenerates SEs (Alwee et al., 2010; Kushairi et al., 2010; Jayanthi et al., 2015; Gomes et al., 2017), making SE a very inefficient process. Moreover, the conversion of SEs into plantlets is often problematic. Regeneration rates are low and many incomplete plantlets lacking a well-developed shoot or root system are formed. The latter seems to be a common problem for different palm species (Gomes et al., 2016). Both Kushairi et al. (2010) and Monteiro et al. (2017) report regeneration rates around 50% for the best treatments.

The combination of limited explant availability and low SE initiation and regeneration rates make it impossible to propagate oil palm at large scale without implementation of a proliferation phase. Proliferation of embryogenic structures does however drastically increase the risk for somaclonal variation (Rival et al., 2013), which is the third main challenge associated with oil palm SE.

### Mantled Abnormality Caused by Tissue Culture Procedures

Soon after the establishment of the first oil palm tissue culture protocols, clones with an aberrant phenotype, the so-called mantled phenotype, were observed. Affected palms are characterized by abnormal flower development and a consequent reduction in oil yield. Because of the high frequency of abnormality, which is only rarely observed in seed-derived palms, the role of tissue culture in the induction of the mantled phenotype became clear, scaring plantation holders to buy more tissue culture planting material. Although tissue culture protocols have been adapted and levels of abnormality have been reduced, the occurrence of the mantled phenotype is still limiting *in vitro* mass propagation of oil palm, with currently ~98% of the planting material consisting of hybrid seeds (Kushairi et al., 2010). The phenotype, cause, and consequences of the mantled abnormality are discussed below.

#### Phenotype of the Mantled Abnormality

**Figure 4:**
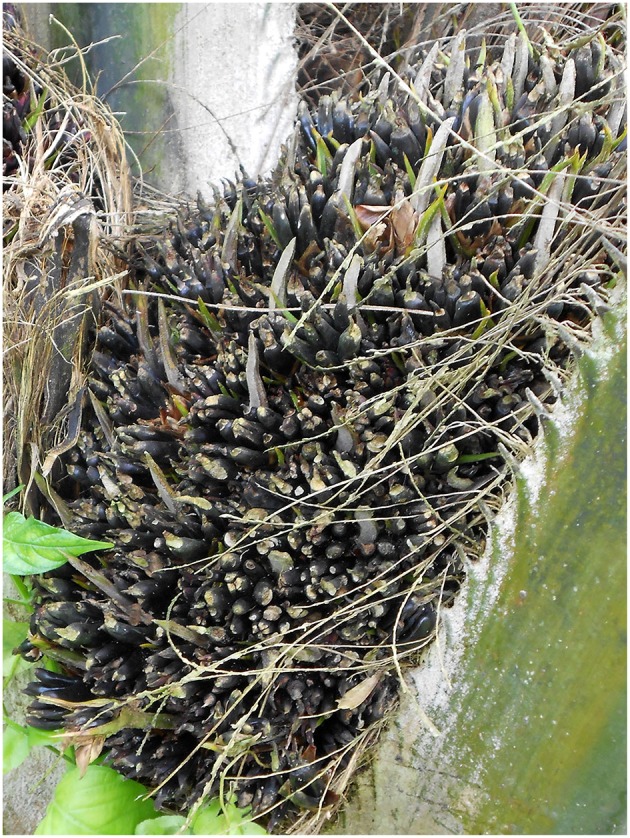
Phenotype of mantled fruits. Mantled fruits are characterized by the presence of extra carpels, arising from a feminization of the staminodes present in female flowers. In slightly mantled palms, mantled flowers might develop into fruits, having a reduced oil content. In more severe cases, flowers are aborted, leading to a total production loss.

During normal oil palm flower development, separate male and female inflorescences are produced in an alternating cycle on the same plant (Adam et al., 2005). Both male and female inflorescences are composed of a central axis, the rachis, containing more than 100 lateral axis or rachillae. Male and female rachillae bear 400–1,500 and 15–30 individual flowers, respectively. All flowers are bisexual in origin. Male flowers consist of three stamens and a rudimentary gynoecium, while female flowers have six rudimentary stamen or staminodes and a gynoecium consisting of three carpels. In both male and female flowers, the reproductive organs are surrounded by three sepals and three petals, both referred to as tepals because of their identical morphology (Adam et al., 2005). Mantled palms are characterized by abnormal floral development, showing similarities to the rare, natural *diwakkawakka* oil palm variant (Adam et al., 2005; Jaligot et al., 2011). Male reproductive organs, namely the stamen in male flowers and staminodes in female flowers, are transformed in carpel-like structures (Figure 4). Resulting male flowers are sterile, while female flowers can be affected with variable severity. In some cases, fruits with reduced oil yield are produced, while in more severe cases flowers are aborted leading to a total loss of oil production (Rival, 2000; Adam et al., 2007).

#### Occurrence and Consequences of the Mantled Abnormality

Since mantled palms are characterized by generative aberrations only, the abnormality can only be visually detected from the moment the trees produce their first inflorescences, meaning around 4 years after field planting. During these first 4 years, the affected palms are taken care of as normal palms, meaning a waste of labor, resources and plantation area, on top of the reduced oil yields (Jaligot et al., 2011).

Rival (2000) reported that out of the 29,415 palms obtained through tissue culture of 127 different clones, 9.7% of palms was affected, with 3.7% only slightly affected, and 6% severely affected with significant impacts on oil yields. However, the frequency of the mantled abnormality and the degree of abnormalities are very variable. There are large differences not only between clones, but also between palms of the same clone and flowers of the same palm (Jaligot et al., 2000). Because of this large variability, the outcome of a certain batch of tissue culture plantlets cannot be predicted, making it impossible for laboratories to guaranty high-quality planting material, limiting their sale.

#### The Epigenetic Origin of the Mantled Abnormality

The mantled abnormality is considered as a form of somaclonal variation. Somaclonal variation is defined as phenotypic variation existing amongst clones that arises during tissue culture procedures. It originates in the extreme stress conditions that are applied during tissue culture in order to disturb the normal growth of the plant tissue. Somaclonal variation can be genetic or epigenetic in origin (Bairu et al., 2011; Miguel and Marum, 2011). Genetic alterations causing somaclonal variation include point mutations, insertions, deletions, changes in ploidy level etc. Alterations of DNA methylation or histone modifications, both leading to a change in gene expression, are possible epigenetic causes of somaclonal variation (Smulders and de Klerk, 2011).

The mantled abnormality is an epigenetic form of somaclonal variation. Its epigenetic origin was concluded after the following observations were made:

1. Partial or complete recovery of the normal phenotype after a period of time in the field
2. Non-Mendelian inheritance of the mantled phenotype
3. The absence of DNA polymorphisms (Rival et al., 1998) or altered ploidy levels (Rival et al., 1997) in mantled palms
4. The presence of global DNA hypomethylation in leaves, inflorescences and callus of mantled trees (Jaligot et al., 2000)
5. The presence of single-sequence methylation polymorphisms and expression polymorphisms in mantled trees (Jaligot et al., 2011).

A role for the gene *EgDEF1*, the oil palm ortholog of the B-class MADS-box genes *APETALA3* from *Arabidopsis thaliana* and *DEFICIENS* from *Antirrhinum majus*, in the development of the mantled abnormality was proposed. This hypothesis was based on the observed similarities between the phenotype of mantled palms and that of mutants of B-class MADS-box genes involved in flower development (Adam et al., 2007; Jaligot et al., 2014). In B-class mutants, petals are transformed into sepals and stamens into carpels. Because of the identical morphology of sepals and petals in oil palm, the former cannot be observed in mantled flowers. However, a conversion of stamens into carpels is clearly present in both male and female mantled flowers, indicating a contribution of a homeotic gene to the mantled abnormality.

Although the expression of *EgDEF1* drastically changes with the phenotype (Jaligot et al., 2014), the involvement of the gene in the establishment of the mantled abnormality was only confirmed in 2015 by Ong-Abdullah et al. who performed epigenome-wide association studies. The methylation status of a LINE retrotransposon denominated *Karma*, located in intron 5 of *EgDEF1*, determines the presence of the mantled phenotype. Dense methylation of *Karma* (*Good Karma* epiallel) predicts normal fruit set, while hypomethylation (*Bad Karma* epiallel), which might arise during stressful tissue culture procedures, leads to alternative splicing of *EgDEF1* and the formation of an incomplete protein. This truncated protein is involved in the establishment of the typical homeotic transformation present in mantled palms (Ong-Abdullah et al., 2015).

#### The Influence of Tissue Culture Procedures on Somaclonal Variation

During *in vitro* culture, plant tissue is exposed to stressful conditions, such as sterilization and wounding, high PGR concentrations, and sometimes sub-optimal medium compositions or environmental conditions. A certain degree of stress might be necessary to obtain cell division and proliferation, but it might on the other hand also induce somaclonal variation (Cassells and Curry, 2001; Fehér, 2015). Several factors related to the tissue culture procedure potentially contribute either alone or combined, to the establishment of the mantled abnormality and are discussed below:

1. *In vitro* propagation method: Some propagation methods trigger a higher risk for somaclonal variation. Generally, the more the organizational structure of the plant is broken down, the higher the risk for mutations. Examples of low risk methods are meristem cultures or methods such as direct organogenesis and embryogenesis in which plant structures are formed directly, without intermediate callus phase. The risk for somaclonal variation drastically increases if a disorganized callus phase or cell suspension phase is included (Bairu et al., 2011), which is the case for oil palm, that currently only can be propagated *in vitro* by indirect somatic embryogenesis.
2. Genotype: Not all genotypes respond in the same way to stress conditions. Some genotypes are less stable and more prone to somaclonal variation than others (Bairu et al., 2011).
3. Starting material: Explants consisting of highly differentiated tissue, such as leaf, flower or root tissue, are more susceptible to somaclonal variation than explants with pre-existing meristems, such as axillary buds or shoot tips (Bairu et al., 2011), that do not have to undergo a dedifferentiation phase. Since immature leaf explants are the preferred explant for oil palm micropropagation, the risk for somaclonal variation is high.
4. Explant *preparation:* Both sterilization of the explant and wounding during excision can cause oxidative stress, which is hypothesized to play an import role in the development of somaclonal variation (Cassells and Curry, 2001).
5. Type and concentration of PGRs: Depending on the plant species, some types and concentrations of PGRs might include a higher risk for somaclonal variation. Mainly auxins and cytokinins, and especially synthetic PGRs, are known as triggers for somaclonal variation. PGRs most likely do not have a direct mutagenic effect but act indirectly through stimulation of rapid disorganized growth (Bairu et al., 2011). As described in section Oil Palm Somatic Embryogenesis Protocols, the oil palm is a recalcitrant plant, requiring high concentrations of PGRs, mainly 2,4-D or picloram, for a prolonged period of time, increasing the risk for somaclonal variation.
6. Number and duration of subcultures*:* Both the limited availability of explants and the necessity to follow the inefficient process of somatic embryogenesis, have led to the implementation of extensive proliferation steps to allow large-scale micropropagation of oil palm. Two types of proliferation can be performed, namely callus proliferation in cell suspension cultures or embryoid multiplication. Independent of the proliferation method used, the higher the number of subcultures, the greater the risk for somaclonal variation (Eeuwens et al., 2002; Rival et al., 2013). Rival et al. (2013) has shown that changes in DNA methylation accumulate in a time-dependent way during cell suspension cultures, increasing the risk for somaclonal variation. Not only the total duration seems to play a role in the development of somaclonal variation, but also the length of the subculture intervals. Eeuwens et al. (2002) have shown that short transfer intervals of 2–4 weeks increase the incidence of the mantled abnormality, while transfer intervals of 8 weeks minimize the risk for somaclonal variation during polyembryoid cultures.

## The Opportunities of Alternative Explant Types

The choice of explant has, together with several other factors, a great influence on the success rate of the regeneration protocol and must be considered well before starting. Large differences in regeneration capacity between different explant types can be observed, which is caused by differences in endogenous hormone concentrations and sensitivity to exogenously applied PGRs (Jiménez, 2005). The regeneration capacity of an explant is mainly determined by its development stage. Generally, juvenile explants are the most responsive.

For startup of oil palm tissue cultures, traditionally immature leaf explants are used, but, as previously described, they are limited in availability and their isolation can strongly damage the ortet. Some alternative explants are available, but their use is currently limited to research purposes. These include immature male and female inflorescences (Teixeira et al., 1994; Guedes et al., 2011; Jayanthi et al., 2015), roots (Eeuwens et al., 2002), mature and immature ZEs (Teixeira et al., 1993; Kanchanapoom and Domyoas, 1999; Thuzar et al., 2011; Balzon et al., 2013; de Carvalho Silva et al., 2014; Gomes et al., 2015, 2016; Wan Nur Syuhada et al., 2016; Monteiro et al., 2017), and young plantlets (Scherwinski-Pereira et al., 2010; Jayanthi et al., 2011). All these types of explants have their own pros and cons, as described below.

Most researchers make use of ZEs as explants. ZEs are popular for research because: (1) they are highly responsive to culture conditions; (2) they are almost free of microorganisms; and (3) they can easily be harvested in large amounts without damaging the ortet (Ree and Guerra, 2015). The disadvantage of ZEs is that their genotype and phenotype differ from the selected ortet, making them less suitable for commercial micropropagation.

Immature inflorescences could form a good alternative to immature leaves as explants, since, in contrast to ZEs, regenerants are identical to the ortet. Furthermore, immature inflorescences are promising explants because of the large number of inflorescences formed by each ortet and the presence of many floral meristems inside the tissue (Guedes et al., 2011). Identical to the isolation of immature leaves, the isolation of immature inflorescences must be performed with care to limit damage to the ortet. Despite the potential immature inflorescences offer, they are, to our knowledge, currently only used for research purposes and not for commercial production of oil palm planting material.

Very few protocols describing somatic embryogenesis from palm root explants are available (Ree and Guerra, 2015). Golden-brown tertiary/quaternary oil palm roots with a diameter of 2 mm have been sterilized and used for callus initiation experiments by Eeuwens et al. (2002). Roots are present in very large amounts, providing an unlimited source of explant tissue. However, the root endosphere and rizosphere, both rich in microorganisms, might make it difficult to obtain sterile explants.

The use of mature female oil palm flowers as explant has not yet been reported, despite this propagation method was already proposed for date palm by Kriaa et al. (2012). The use of mature female flowers could provide some advantages over other explant types. Firstly, mature inflorescences can be isolated without harming the ortet. At maturity, the inflorescences are fully emerged and can be easily chopped off the palm tree. Secondly, female flowers are continuously available. Each tree produces on average two new leaves per month, each accompanied by an inflorescence meristem in its axil. A female inflorescence is composed of more than 2,000 flowers. The number of explants that could be isolated from only a single ortet is thus very high compared to leaf explants. A possible difficulty associated with mature flowers could be the higher risk for contamination compared to immature explants, which are not exposed to the external environment.

Both the degree and timing of reaction might depend on the explant used. Available data for different explant types are summarized in Table 2. Generally, ZEs give the fastest reaction and highest reactivity. Variable reaction percentages have been reported for both immature leaf and inflorescence explants, representing the genotype dependency of callogenesis. Immature leaf explants give slightly lower reaction percentages than immature inflorescences, but they tend to react faster. Each explant thus has its own pros and cons.

**Table 2:** Pros and cons of different explants suitable for oil palm somatic embryogenesis.

**Explant type**	**Pros**	**Cons**	**Primary callus formation**	**Embryogenic callus** **formation**	**Percentage of explants forming callus**	**References**
Immature leaves	Established protocol	Ortet damaged upon harvest; Limited availability	8 w	21 w	Up to 52%	Alwee et al., 2010; Corrêa et al., 2016; Gomes et al., 2017
Zygotic embryos	High reactivity; Free of microorganisms; No damage to ortet upon harvest; High availability	Unknown genotype	6 w	12 w	Up to 97.5%	Balzon et al., 2013; de Carvalho Silva et al., 2014; Ree and Guerra, 2015
Immature inflorescences	Higher availability than leaf explants	Ortet damaged upon harvest	8 w	42 w	Up to 72%	Teixeira et al., 1994; Guedes et al., 2011; Jayanthi et al., 2015
Roots	No damage to ortet upon harvest; High availability	Contamination?	–	–	–	
Mature inflorescences	No damage to ortet upon harvest; High availability	Contamination?	–	–	–	

*The pros and cons of different explant types, as well as the number of weeks necessary to obtain primary and embryogenic callus and the callus formation rate of each explant type, are listed. w, number of weeks after explant inoculation on callus induction medium*.

## The Potential of Alternative Propagation Techniques

Although the oil palm is currently only propagated *in vitro* by indirect somatic embryogenesis, some less known micropropagation techniques, such as direct SE and the reversion of floral meristems, could theoretically be applied to oil palm as well and are briefly described below.

### Direct Somatic Embryogenesis

SEs cannot only be induced from EC, but also directly from the explant. This phenomenon, called direct embryogenesis, has been reported by Jayanthi et al. (2011), who obtained SEs directly on cotyledonary nodes from germinated oil palm ZEs. A combination of five PGRs was used, namely the auxins 2,4-D (40 μM), NAA (40 μM), and 2,4,5-trichlorophenoxyacetic acid (2,4,5-T; 10 μM), and the cytokinins 6-benzylaminopurine (BA; 10 μM) and thidiazuron (TDZ; 10 μM). To date, this is the only report of direct SE in oil palm. Since direct SE is less susceptible to somaclonal variation, exploring the possibility to induce direct SE from other explant types could contribute to the development of an improved propagation method.

Two main methods for induction of direct SE could be examined. The most straightforward method relies on varying culture medium composition and environmental conditions, but genetic techniques can also be used for induction of direct SE. During the induction of SE, several genes, mainly stress-related genes, PGR-related genes and genes encoding transcription factors, are upregulated, triggering different signaling processes (Guan et al., 2016; Méndez-Hernández et al., 2019). Overexpression of some of these genes, including *BABY BOOM* (BBM; Boutilier et al., 2002), *WUSCHEL* (Zuo et al., 2002) and *LEAFY COTYLEDON1* (Lotan et al., 1998) conferred direct SE formation in different plant species. Although some efforts have been made in the identification of oil palm genes involved in SE (Le et al., 2011; Ooi et al., 2012; Syariyanto et al., 2018), to date no reports on the overexpression of any embryogenesis related genes have been made, indicating an important research field for the future.

### Reversion of Inflorescence Meristems

The oil palm only has one vegetative meristem but an infinite number of generative meristems. Each new leaf is accompanied by an inflorescence meristem and each inflorescence is composed of several thousands of flower meristems. Conversion of these generative meristems into vegetative meristems could allow the proliferation of oil palm by meristem cultures. This idea has been described by Staritsky (1970) and has been successfully applied to coconut palm (Abahmane, 2013; Zayed et al., 2016). Floral reversion, including both flower and inflorescence reversion, involves a switch from floral development back to vegetative development (Tooke et al., 2005). This reversion might be incomplete, with some parts of the flower replaced by leaves, or complete, with vegetative propagation occurring after flowering. Floral reversion is strongly associated with environmental conditions, mainly with temperature and daylength. Mostly conditions opposite to flowering induction conditions can induce floral reversion. A well-known example of floral reversion is the *Impatiens balsamina* cultivar Dwarf Bush Flowered, which is a short-day plant that reverts to vegetative growth when transferred to long-day conditions (Tooke et al., 2005). Although floral reversion is a very challenging research field, it could increase our knowledge about oil palm flowering mechanisms and, if conversion could be achieved, it could lead to the development of a total new propagation technique.

## Increasing Somatic Embryo Initiation and Regeneration Efficiency

### Improving SE Initiation Rates by Early Detection of Embryogenic Cells

Only a few of the callus cells that are formed during the callus induction phase are able to regenerate SEs. Early identification of these cells would mean an increased efficiency of oil palm SE, associated with a reduction in labor and costs. In some cases, embryogenic cells can be distinguished from non-embryogenic cells based on morphological characteristics solely. Embryogenic cells generally resemble meristematic cells: they are small, isodiametric cells with a dense cytoplasm, large nuclei, evident nucleoli, small vacuoles, thick cell walls and a high metabolic activity (Quiroz-Figueroa et al., 2006). However, visual screening for embryogenic cells is a subjective and time-consuming method. The use of expression markers is considered as one of the most effective techniques for detection of EC (Syariyanto et al., 2018). Together with the sequencing of the oil palm genome, transcriptome data from different tissue types and experimental conditions have become available (Singh et al., 2013b). Based on these data, different research groups are studying oil palm gene expression and regulation (Low et al., 2008; Bourgis et al., 2011; Xia et al., 2014; Syariyanto et al., 2018). Several candidate genes with differential expression between embryogenic and non-embryogenic callus have been identified, such as *EgIAA9*, a putative Aux/IAA gene (Ooi et al., 2012), *Eg707*, encoding an unknown protein that is likely involved in abscisic acid biosynthesis (Le et al., 2011) and *ILR1-like 1*, encoding an IAA-amino acid hydrolase (Syariyanto et al., 2018). Further research into these and other candidate genes, might allow the development of an efficient screening method for identification of palms highly responsive to somatic embryogenesis.

### Methods to Improve Oil Palm SE Regeneration Rates

In nearly all oil palm regeneration protocols published, initiation and maturation of SEs are combined in a single phase, using the same culture medium (see Table 1). However, inclusion of a separate maturation phase could increase the quality of SEs and lead to higher germination rates. The maturation of SEs is very similar to that of zygotic embryos. The embryo undergoes biochemical and morphological changes: storage material is deposited and desiccation tolerance is acquired (Ochatt and Revilla, 2016). Upon lack of an appropriate maturation treatment, many SEs do not develop normally or do not germinate at all. ABA is the most used PGR for embryo maturation. In certain species, especially in conifers, the addition of ABA to the culture medium stimulates maturation. In other species ABA is often applied to prevent precocious germination (von Arnold, 2008). Limited information about the effect of ABA on germination of oil palm SEs is available. Three different maturation treatments, namely 0.46 μM kinetin, 2.32 μM kinetin, and 0.46 μM kinetin + 0.19 μM ABA, were compared by Sumaryono et al. (2008), whereby the addition of ABA lead to a significant increase in mature SEs after 6 weeks in maturation medium. Another way to improve the quality of SEs is to lower the osmotic potential of the maturation medium, mimicking the desiccation period of normal seeds. Lowering the osmotic potential of the medium can for example be obtained by increasing the sugar or macronutrient concentration of the culture medium, or by the addition of polyethylene glycols (von Arnold et al., 2002). Cold treatments can induce a similar effect (Jiménez, 2005). No desiccation treatments have been reported for oil palm, but it has successfully been applied in date palm propagation procedures (Othmani et al., 2011). When EC were incubated for 12 h in petridishes containing three Whatman filter papers to mimic drought stress, the number of mature SEs formed was significantly higher than in control treatments.

Regeneration of oil palm SEs is traditionally performed on semi-solid media. However, commercial micropropagation based on semi-solid media is cumbersome as it is very labor-intensive and requires a lot of space, leading to high production costs. Alternatively, TIS can be used for regeneration of SEs. A comparison between RITA® bioreactors, Twin-Flask type of bioreactors and semi-solid media on oil palm SE regeneration was performed by Gomes et al. (2016). Torpedo-shaped SEs were inoculated in the three different systems and the effect of the culture system on regeneration was determined after three 30-day subcultures. The number of plantlets regenerated was similar for both bioreactors and significantly higher than for the semi-solid system. However, when looking at the fresh biomass increment and height of the plant, the Twin-Flask system was superior to the RITA® bioreactor. Another benefit of both bioreactor systems compared to semi-solid systems, is the increased formation of secondary SEs, which provide a source of plantlet proliferation.

To overcome low rooting rates of SE-derived plantlets, an *in vitro* rooting stage in which exogenous auxins are applied, can be introduced. An extensive rooting experiment on incomplete SE-derived oil palm plantlets was performed by Gomes et al. (2015). Different indole-3-butyric acid (IBA) and NAA concentrations, the presence or absence of AC and three different culture systems (semi-solid medium, stationary liquid medium, and a double-phase system consisting of a liquid layer on top of a semi-solid layer) were compared. A concentration of 53.7 μM of IBA significantly increased the rooting percentage as well as the number of roots formed per plantlet. Optimal results were obtained within the double-phase system, with root induction percentages up to 92.9% after 150 days. The superior performance of the double-phase system might be explained by the increased availability of water and nutrients due to the presence of a liquid medium portion compared to the semi-solid system, and the reduced risk for hyperhydricity compared to the stationary liquid system. The addition of AC was detrimental for root induction, although it stimulated root elongation. A rooting phase of at least 90 days should be incorporated in the protocol for optimal rooting of non-rooted shoots.

## Eliminating the Influence of Somaclonal Variation on Clonal oil Palm Production

**Figure 5:**
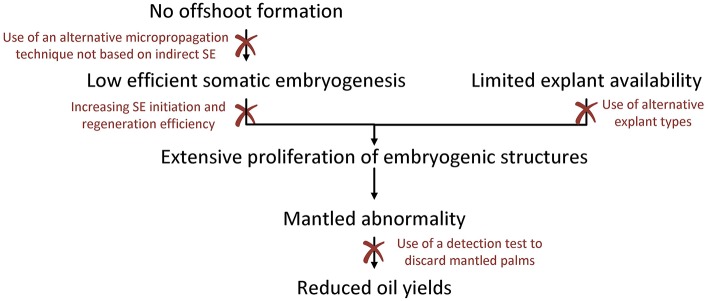
Challenges associated with oil palm tissue culture. Because the oil palm does not form any offshoots, tissue culture is based on indirect somatic embryogenesis, a process with very low efficiencies. The low efficiency of somatic embryogenesis, combined with the limited availability of immature leaf explants, necessitate the extensive proliferation of embryogenic structures to allow mass propagation of oil palm. However, proliferation of embryogenic structures strongly increases the risk for somaclonal variation, including the mantled abnormality, which is limiting oil yields. This sequence of events accumulating in the formation of mantled palms with reduced oil yields, can be interrupted at several points. Interventions such as an increased efficiency of somatic embryogenesis, the use of alternative micropropagation methods not based on somatic embryogenesis or the use alternative explant types with higher availability could limit the incidence of somaclonal variation, while the early detection of mantled palms could avoid the planting of mutated trees in the field. All these interventions provide interesting fields for future research and could contribute to the establishment of an improved oil palm micropropagation protocol not hampered by somaclonal variation.

Although the SE protocol has been adapted in the past to reduce the incidence of somaclonal variation to a minimum, the mantled abnormality is still an important factor limiting the large-scale production and sales of tissue culture oil palm planting material. The occurrence of the mantled phenotype is caused by several factors associated with the indirect SE protocol, of which the extensive proliferation of embryogenic structures might be the most important one. Due to the limited access to immature leaf explants and the low SE initiation and regeneration rates, it is currently impossible to proliferate oil palm at large scale without performing EC or embryoid proliferation. All challenges involved in oil palm tissue culture as described in this paper are thus inherently linked to each other, accumulating in the occurrence of the mantled phenotype, the final reason oil palm tissue culture is not widely implemented. An overview of the challenges and how they are connected is shown in Figure 5.

Intervening in one or more steps of the series of challenges, might lead to the large-scale production of tissue culture plantlets without being hampered by somaclonal variation. Generally, there are two types of possible interventions: (1) those aiming to limit the incidence of somaclonal variation, and (2) those aiming to detect mantled palms in an early stage, as to avoid they are sold to plantation holders. The former includes interventions such as an increased efficiency of SE, the use of alternative micropropagation methods not based on SE or the use of alternative explant types with higher availability. The latter includes the development of a rapid and affordable detection technique for early detection of mantled palms. Both types of interventions are discussed below.

### Limiting the Incidence of Somaclonal Variation

In this review several ways to increase the efficiency of the oil palm micropropagation protocol have been put forward, including the use of alternative explant types, the development of a micropropagation method not based on indirect SE or the increase of SE initiation and regeneration rates. As shown in Figure 5, these strategies could not only directly improve the efficiency of the tissue culture protocol, but also indirectly by limiting the risk for somaclonal variation. All these strategies namely reduce the need for proliferation of embryogenic structures, a major factor involved in the development of the mantled abnormality.

### Detecting the Mantled Mutation in an Early Stage

Another way to limit the impact of the mantled abnormality on oil palm tissue culture, is the detection of affected plantlets before they are being planted in the field. In this way, no labor, plantation area and resources are spilled to mutated plantlets and a yield reduction is avoided. By incorporating a detection step in the propagation protocol, the delivery of high-quality, mantled-free planting material can be assured.

Although global hypomethylation, single-sequence methylation polymorphisms and expression polymorphism were already detected several years ago, none of these were useful as a detection marker for early identification of mantled palms, because of the very high individual- and genotype-dependent variation in methylation and expression levels observed (Jaligot et al., 2011). It was only in 2015, upon the discovery of the role of *Karma* (see above), that a detection test was developed by Orion Biosains, a subsidiary of Orion Genomics. The so-called SureSawit™ *KARMA* test allows for the detection of mantled palms at the nursery stage. With the use of a collection kit, a leaf punch is taken and sent to the laboratory for analysis^2^. There, leaf DNA is extracted and treated with bisulfite. Bisulfite converts cytosine into uracil but leaves 5-methylcytosine intact. In this way, the methylation status of *KARMA* can be easily detected with a PCR assay (Ong-Abdullah et al., 2016).

A fast increase in production of tissue culture plant material was expected to be associated with the development of this test, but, as far as we know, the production is still on a low level. Several hypotheses for this can be put forward: (1) The cost of the SureSawit™ *KARMA* test might be too high to test all tissue culture plantlets, increasing the cost price of the planting material above the budget of the farmers; (2) Plantation holders would possibly like to see the performance of the test with their own eyes, before implementing it to large scale. This might take several years, namely until the first tested palms are flowering and it can be visually confirmed that they do not exhibit the mantled phenotype; (3) The test might not perform as well as expected, for example due to large genotype-dependent variation, with lots of false negatives popping out. More research is needed to validate the SureSawit™ *KARMA* test.

An efficient detection technique for early identification of mantled plants, seems inevitable to increase the demand of tissue culture plant material. Therefore, the possibilities to optimize current detection technique should be examined. Also, the efficiency of other methylation detection techniques, not based on bisulfite treatment, on the early detection of the mantled abnormality can be tested. Bisulfite treatment is the most common technique for studying DNA methylation but has several drawbacks, including the inclusion of a costly and time-consuming sample preparation step, and the risk for DNA degradation by the harsh reaction conditions applied (Flusberg et al., 2010). Several alternative methylation detection techniques have been developed, such as the single-molecule real-time (SMRT) sequencing method described by Flusberg et al. (2010). Fluorescently labeled nucleotides are incorporated in the complementary strand by a DNA polymerase. The fluorescent pulses give information about the polymerase kinetics and allow the direct detection of methylated nucleotides. To reduce the occurrence of false negative test results, it might be interesting to combine two different detection techniques. Although this would give more certainty about the plant phenotype, the cost price to test a plantlet would increase. Also, further research into the mode of action of *Karma* in the establishment of the mantled phenotype could deliver important information for optimization of a detection test.

## Concluding Remarks

To date, the large-scale production of oil palm plantlets by tissue culture is limited because of several difficulties associated with the propagation procedure, which is based on indirect SE starting from immature leaf explants. In this review, several research directions have been put forward, that aim to allow mass production of tissue culture planting material while limiting the risk for somaclonal variation. Some of the proposed techniques, e.g., direct SE and floral reversion, might be more challenging and less likely to be successful than others, but the most challenging techniques could also provide the most progress toward a more efficient and reliable oil palm micropropagation protocol. To allow mass propagation of oil palm by tissue culture, most likely a combination of several different adaptations to the protocol will be needed, indicating the importance of the investigation of several of the proposed research topics instead of focusing on one technique. For example, an increase in SE induction and/or regeneration efficiency alone could increase the number of plantlets produced from a single isolation of immature leaves and could thus reduce the need for a proliferation phase but it will never allow the elimination of callus or embryoid proliferation completely. Somaclonal variation will thus still be present to a certain amount. On the other hand, if an increase in SE induction and/or regeneration efficiency could be combined with a performant new detection technique, mass propagation of reliable oil palm tissue culture plantlets could be achieved. Tissue culture planting material provides many advantages over hybrid seeds in increasing oil yield on the current cultivated area. Since the demand for sustainable palm oil keeps growing, it is important to keep investing in the optimization of oil palm tissue culture procedures.

## Author Contributions

The manuscript was prepared by SW and reviewed by DI and LM.

### Conflict of Interest Statement

SW and LM were employed by the company Deroose Plants NV. The remaining author declares that the research was conducted in the absence of any commercial or financial relationships that could be construed as a potential conflict of interest.

## Footnotes

^1^https://apps.fas.usda.gov/psdonline/app/index.html#/app/home

^2^https://www.orionbiosains.com/products/services/karma
